# Role of Sirtuin 3 in Degenerative Diseases of the Central Nervous System

**DOI:** 10.3390/biom13050735

**Published:** 2023-04-24

**Authors:** Haofuzi Zhang, Shuhui Dai, Yuefan Yang, Jialiang Wei, Xin Li, Peng Luo, Xiaofan Jiang

**Affiliations:** 1 Department of Neurosurgery, Xijing Hospital, Fourth Military Medical University, Xi’an 710032, China; 2 Institute of Neurosurgery of People’s Liberation Army of China (PLA), PLA’s Key Laboratory of Critical Care Medicine, Xijing Hospital, Fourth Military Medical University, Xi’an 710032, China; 3 National Translational Science Center for Molecular Medicine and Department of Cell Biology, Fourth Military Medical University, Xi’an 710032, China; 4 Department of Biomedical Engineering, Fourth Military Medical University, Xi’an 710032, China; 5 Department of Health Service, Fourth Military Medical University, Xi’an 710032, China; 6 Department of Anesthesiology, Xijing Hospital, Fourth Military Medical University, Xi’an 710032, China

**Keywords:** Sirtuin 3, mitochondrial function, Alzheimer’s disease, Parkinson’s disease, Huntington’s disease, amyotrophic lateral sclerosis, multiple sclerosis, neurodegenerative diseases

## Abstract

An NAD^+^-dependent deacetylase called Sirtuin 3 (Sirt3) is involved in the metabolic processes of the mitochondria, including energy generation, the tricarboxylic acid cycle, and oxidative stress. Sirt3 activation can slow down or prevent mitochondrial dysfunction in response to neurodegenerative disorders, demonstrating a strong neuroprotective impact. The mechanism of Sirt3 in neurodegenerative illnesses has been elucidated over time; it is essential for neuron, astrocyte, and microglial function, and its primary regulatory factors include antiapoptosis, oxidative stress, and the maintenance of metabolic homeostasis. Neurodegenerative disorders, such as Alzheimer’s disease (AD), Parkinson’s disease (PD), Huntington’s disease (HD), amyotrophic lateral sclerosis (ALS), and multiple sclerosis (MS), may benefit from a thorough and in-depth investigation of Sirt3. In this review, we primarily cover Sirt3’s role and its regulation in the nerve cells and the connection between Sirt3 and neurodegenerative disorders.

## 1. Introduction

Neurodegenerative disorders are described by a progressive loss of neurons and by protein deposition, which are present in Alzheimer’s disease (AD), Parkinson’s disease (PD), Huntington’s disease (HD), amyotrophic lateral sclerosis (ALS), multiple sclerosis (MS), and so on [1,2]. As the population ages, neurodegenerative disorders put a significant burden on society and families while reducing the patients’ quality of life. The pathological mechanisms of neurodegenerative diseases are complex and diverse, and, particularly, the molecular mechanisms underlying the majority of neurodegenerative diseases are not fully understood. On account of these problems, clinical examination items are limited, which makes early diagnosis and treatment difficult [3]. As a result, it is crucial to determine efficient treatment and prevention methods to explore the mechanisms and potential targets of neurodegenerative diseases.

Recent research on the processes behind neurodegenerative diseases has revealed that neuronal death is influenced by a number of factors, including excitatory toxicity, mitochondrial malfunction, inflammation, oxidative stress, and apoptosis [4]. Although the mechanism involves a number of aspects, mitochondrial dysfunction and abnormal energy metabolism have become recognized as early pathological phenomena and have drawn widespread attention [5]. The abnormal mitochondrial function described above triggers a series of interactions in an injurious process, leading to, or accelerating, the onset or exacerbation of neurodegenerative diseases. An NAD^+^-dependent deacetylase called Sirt3 participates in mitochondrial metabolic processes, including energy generation [6], the tricarboxylic acid cycle [7], and oxidative stress [8]. It has the functions of antioxidative stress [9], antiapoptosis [10], and the maintenance of metabolic homeostasis [11]. The primary source of the energy supply for the brain is the decomposition of glucose, which generates ATP. Following mitochondrial damage, insufficient cellular energy production can easily lead to impaired cognitive function. By deacetylating various ATP synthase subunits, Sirt3 supplies energy to the brain in order to maintain its day-to-day activities [12].

In conclusion, because the central nervous system (CNS) has a high rate of metabolism and a small energy reserve, neurological diseases can involve varying degrees of mitochondrial dysfunction. Since Sirt3 has drawn extensive attention owing to its important part in oxidative stress and energy metabolism, this article reviews the progress of the research on the relationship between Sirt3 and the degenerative diseases of the CNS.

## 2. Molecular Structure and Function of Sirt3

### 2.1. Sirtuin Family and Sirt3

**Figure 1:**
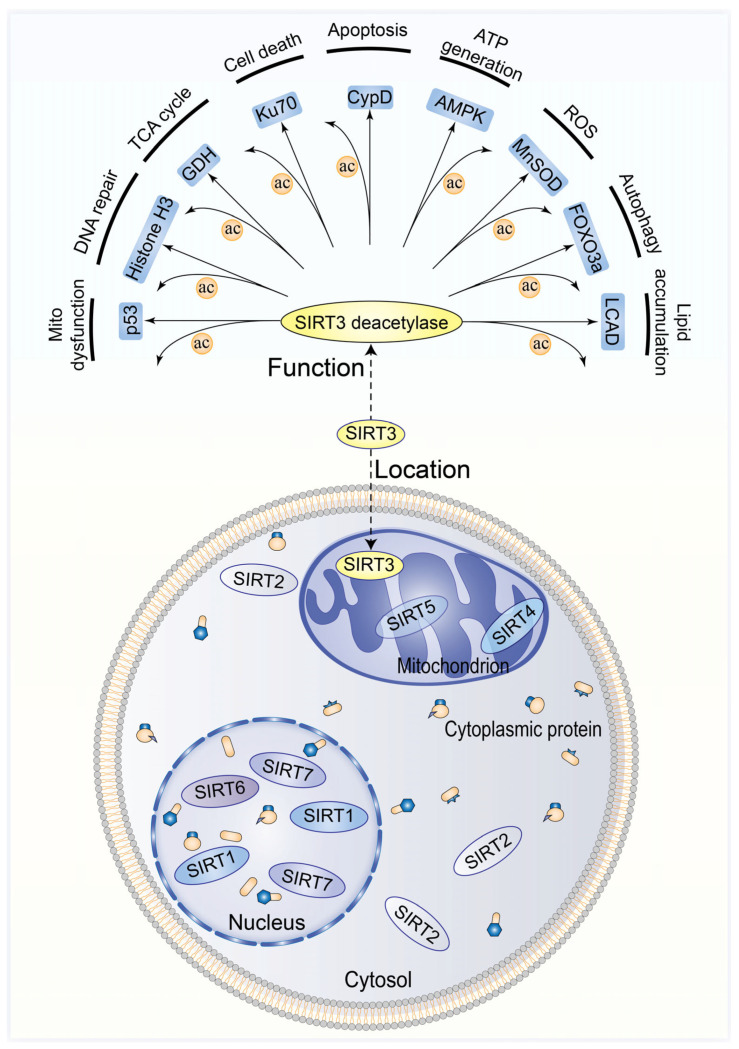
Sirtuin family and the function of Sirt3 in oxidative stress and mitochondrial metabolism regulation. AMPK, AMP-activated protein kinase; TCA, tricarboxylic acid; MnSOD, manganese superoxide dismutase; ROS, reactive oxygen species; CypD, cyclophilin; HMGCS2, 3-hydroxy-3-methylglutaryl CoA synthase-2; LCAD, long-chain acyl-CoA dehydrogenase; AceCS2, acetyl-CoA synthetase 2; GDH, glutamate dehydrogenase; FOXO3a, forkhead-box-containing protein class O3a; PGC-1 α, peroxisome-proliferator-activated receptor gamma coactivator-1 alpha.

The sirtuin family is a group of highly conserved nicotinamide adenine dinucleotide (NAD^+^)-dependent protein deacetylases that occur during the aging process of a variety of organisms, such as worms, yeasts, humans, fruit flies, and mice [13]. Seven species of the mammalian sirtuin family have been identified, sirtuin 1–7 (Sirt1-7) [14]. The nucleus is home to Sirt7, Sirt1, and Sirt6; meanwhile, the cytoplasm is the primary location of Sirt2, and the mitochondria are the primary location of Sirt5, Sirt3, and Sirt4. Sirtuins (1–7) carry out several enzymatic processes in the presence of NAD^+^, including deacetylation, ADP-ribosylation, demalonylation, depalmitoylation, desuccinylation, deglycosylation, and demyristoylation [15]. Additionally, sirtuins are crucial in interacting with many other pathways that regulate aging, including the mammalian target of rapamycin (mTOR) pathways [16] and insulin-forkhead box protein O (FOXO) (Figure 1) [17].

**Figure 2:**
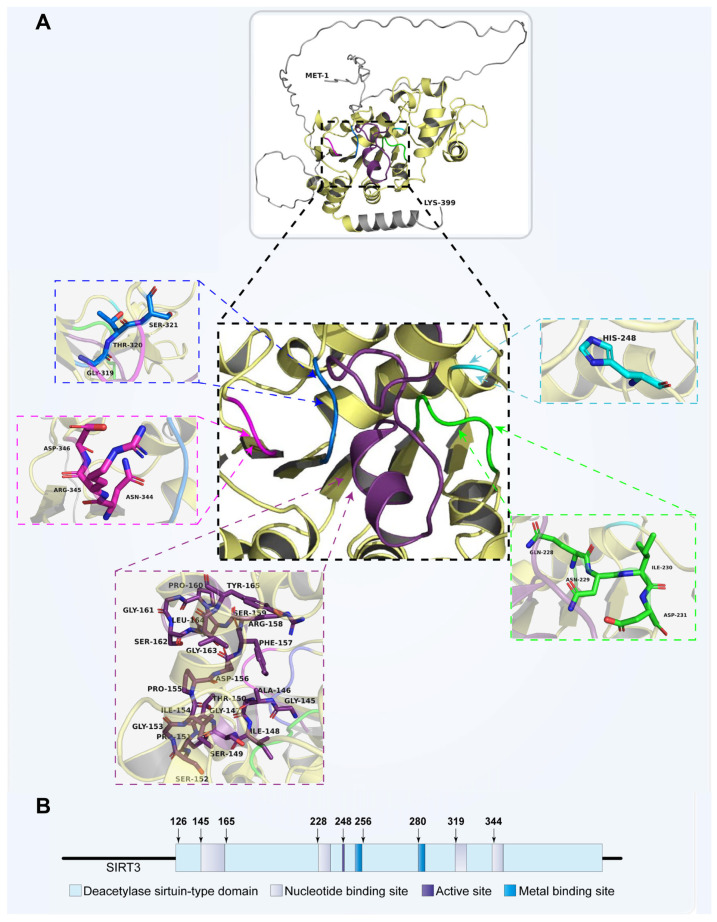
Spatial structure model of Sirt3 and its functional sites. (**A**) The NAD^+^-binding Rossmann fold in Sirt3’s conserved catalytic core is composed of numerous inverted classical open α/β structures. Following this is a groove with multiple loops that serve as a connecting cleft between the acetylated peptide substrate and NAD^+^, followed by a substrate-binding site. Pale yellow: deacetylase sirtuin-type domain (126–382); violet purple: NAD^+^-binding site (145–165); green: NAD^+^-binding site (228–231); marine: NAD^+^-binding site (319–321); magenta: NAD^+^-binding site (344–346); cyan: active site (248). (**B**) Sirt3 has binding sites for NAD^+^ and Zn^2+^ and has an active site that acts as a proton acceptor.

Sirt3 (PBD code: 3GLS) is typically found in tissues and organs with high metabolic rates, including the liver [18], brain [19], heart [20], and brown adipose tissue [21]. It features a conserved enzymatic core and two domains: a large Rossmann fold domain that binds NAD^+^ and a tiny domain formed by the two insertions of the big domain attaching to a zinc atom. The acetylated peptide substrate binds to the cleft between the two domains. Sirt3 substrates include a few important enzymes known to play a role in mitochondrial energy generation. The cofactor-binding pocket can be divided into three regions: the catalytic center, the adenine ribose moiety of NAD^+^, and the nicotinamide ribose moiety (Figure 2) [22,23]. The regulation of oxidative stress and mitochondrial metabolism is greatly influenced by Sirt3 [24], the major mitochondrial deacetylase found in the mitochondrial matrix (Figure 1).

### 2.2. Molecular Function of Sirt3

Sirt3 acts as the main deacetylating enzyme in the mitochondria. The NADH and NAD^+^ content regulates its enzymatic activity. The currently identified Sirt3 protein targets consist of long-chain acyl-CoA dehydrogenase (LCAD) [25], isocitrate dehydrogenase 2 (IDH2) [26], glutamate dehydrogenase (GDH) [7], succinate dehydrogenase A (SDHA) [27], NADH dehydrogenase [28], pyruvate dehydrogenase A (PDHA) [29], ATP synthase [24], acetyl-CoA synthase 1 (ACSS1) [30], ornithine transcarbamylase (OTC) [31], mitochondrial ribosomal protein L10 (MRPL10) [32], cyclophilin D (CypD) [33], mitochondrial chaperone Hsp10 [34], and mitochondrial Lon peptidase 1 (LONP1) [35]. Hence, in order to regulate almost all the signaling pathways related to cellular metabolism in the body, Sirt3 is engaged (Figure 1).

Sirt3 is also involved in the repair of mitochondrial DNA damage, the prevention of oxidative-stress-induced apoptosis, and the preservation of mitochondrial integrity. By increasing the levels of catalase (CAT) and manganese superoxide dismutase (MnSOD) and by deacetylating the important transcription factor forkhead-box-containing protein class O3a (FOXO3a), it can also decrease oxidative stress [36]. Additionally, it has been demonstrated that Sirt3 is translocated into the nucleus and functions as a histone deacetylase to control the epigenetic regulation of several genes [37].

Therefore, the molecular functions of Sirt3 in the human body are as follows: (1) stabilizing genes; (2) regulating the respiration of the mitochondria; (3) regulating adenosine triphosphate (ATP) formation and balancing the redox state of cells by regulating metabolism to stabilize the energy of cells and the activity of enzymes; (4) regulating fatty acid β oxidation; (5) antioxidant effects, including taking part in most of the oxidative stress processes and preventing excessive reactive oxygen species (ROS) production in the mitochondria; and (6) maintaining the stable morphology and structure of the mitochondria and taking part in the proliferation, metabolism, survival, aging, and lifespan of the organs (Figure 1).

## 3. Cellular Function of Sirt3 in the CNS

The metabolic rate of the CNS is high and there is almost no energy reserve; therefore, the diseases of the CNS involve varying degrees of mitochondrial dysfunction. With the increased attention being paid to Sirt3 in recent years, more and more research results have proven that Sirt3 has a unique role and potential in the CNS. Among the numerous causes of neural injuries, such as cerebral ischemia [38], traumatic brain injuries (TBIs) [39], tumors [40], and neurodegenerative diseases [41], oxidative stress, disturbances in energy metabolism, and apoptosis are the cytological basis of these injuries. Mitochondrial dysfunction is an underlying cause of cell death, and Sirt3, a deacetylase found in the mitochondria that regulates cellular energy metabolism, is undoubtedly required for metabolic adaptation to various physiological threats [42].

At the level of molecular regulation, mitochondrial Sirt3 is normally a SUMOylated protein, and the activity of deacetylase is temporarily inhibited. Upon stimulation by external damaging factors, the upstream regulatory molecule, sentrin-specific protease 1 (SENP1), deSUMOylates Sirt3 to make it deacetylate the proteins in the mitochondria [42], thereby producing specific effects. This is the molecular basis for Sirt3 to play a regulatory role in the cells. The following section describes Sirt3 in the neurons, astrocytes, and microglia.

### 3.1. Sirt3 and Neurons

The most fundamental functional and structural components of the CNS are the neurons, which are crucial for synaptic signaling. They can receive stimuli and generate and transmit excitation. However, neurons lose their ability to regenerate during development, which is the physiological basis for the often irreversible nature of nerve damage [43]. Sirt3 acts as a prosurvival factor and is essential for the protection of the neurons from physiological threats and pathological damage [44].

**Figure 3:**
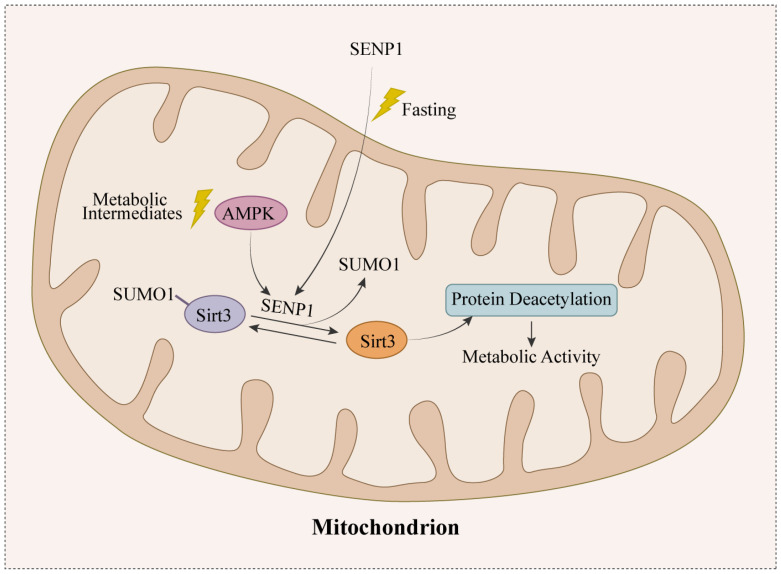
The cross-regulation of Sirt3 and AMPK.

To cope with harmful factors, the expression of Sirt3 in the neuronal mitochondria rises [45,46], enhancing, to some extent, the antioxidant capacity of the neurons, as well as reducing mitochondrial dysfunction [47]. What is known is that there is crosstalk between Sirt3 and the cytoprotective AMP-activated protein kinase (AMPK) signaling pathway [48]. In cerebral ischemia, the low-glucose state activates the SENP1-Sirt3 signaling pathway through nonclassical AMPK signaling [49], while the accumulation of glucose intermediate metabolites due to mitochondrial dysfunction, on the other hand, inhibits the classical cytoprotective signaling pathway AMPK-SENP1-Sirt3 (Figure 3). It has been shown that, in the subacute phase of cerebral ischemic injuries, the level of SENP1 in the neurons decreases, leading to a decrease in Sirt3 activity and an increase in mitochondrial protein acetylation, a process that causes oxidative stress and mitochondrial dysfunction, leading to an increase in reactive oxygen species (ROS) and a decrease in the ATP content [50]. In various CNS injuries, by modulating Sirt3 and thus stimulating the AMPK signaling pathways, it is possible to defend against ischemic hypoxia [51] and oxidative damage [52,53], to rescue mitochondrial ATP production [54], to improve defects in synaptic plasticity, and to reduce the hyperexcitability of the neural networks [47]. Furthermore, the protective effects of Sirt3 against brain injury may be achieved through processes such as the regulation of neuronal Ca^2+^ homeostasis [55], the inhibition of ROS accumulation, the activation of mitochondrial biosynthesis [56], and the reduction of mitochondrial dysfunction [57].

Overall, energy depletion and oxidative stress contribute to the development of neural injury, and the critical role of Sirt3 in resisting ROS and maintaining energy homeostasis through mitochondrial functional protection is important for intervening in neuronal death.

### 3.2. Sirt3 and Astrocytes

The largest of the glial cells and the most prevalent type of cell in the mammalian brain are the astrocytes. They have a crucial role in the formation of the blood–brain barrier (BBB) and in the division and maintenance of the nerve cells. They can also respond to inflammatory signals and participate in regulating multiple life processes of the CNS under physiological and pathological conditions. When brain damage caused by various problems occurs, it is often accompanied by obvious BBB destruction and an inflammatory reaction [58], which is related to mitochondrial dysfunction to a certain extent and is regulated by Sirt3.

Precise Sirt3 overexpression in the astrocytes at an ischemic injury site alleviates the injury [59], suggesting that Sirt3 may serve as a key regulator in BBB physiology through the astrocytic function [60]. It has been highlighted that nicotinamide mononucleotides (NMNs) stimulate oxidative phosphorylation in the mitochondria while improving numerous pathologies in mouse disease models. In primary mouse astrocytes, a Sirt3 knockdown reverses the impact of NMNs through the inhibition of energy production [61]. In the inflammatory response, the lipopolysaccharide (LPS)/interferon-γ stimulation of the astrocytes reduces mitochondrial dysfunction and oxidative-stress-induced cell death through the regulation of Sirt3 [62]. In addition, as a component of traditional Chinese medicine that has a therapeutic effect, the trefoil protein (TLB) can activate the Sirt3/SOD2 signaling pathway in the astrocytes [63], which also plays a considerable neuroprotective role, making Sirt3 a promising target.

To summarize, astrocytes may be implicated in a number of neuropathological processes. The research points out that Sirt3 is critical to these processes.

### 3.3. Sirt3 and Microglia

The microglia are the only cells in the neural tissue that are derived from the mesoderm, and they play a part in stress responses and inflammation. They are also the CNS’s main immune cells with phagocytic activity. Brain injury causes the microglia to alter their morphology and to release pro- and anti-inflammatory mediators [64]; therefore, inhibiting overactive microglia is thought of as a potential therapeutic strategy, in which Sirt3 plays an important role.

Microglial activation and neuroinflammatory cytokine levels are regulated in a Sirt3-dependent way [65]. Sirt3-silenced microglia show significant cytotoxicity and changes in cell morphology when they are exposed to an injury stimulus [66], and the adverse effects of overactivated microglia are strongly related to oxidative stress [67]. Studies have shown that, after hypoxia, the expression of Sirt3 in the microglia is upregulated, which inhibits excessive cell activation and alleviates the damage of hypoxic stress to the CNS [68]. Additionally, Sirt3 protects the neural stem cells (NSCs) from the damaging effects of oxidative stress brought on by microglial activation [69]. Moreover, treatment with gastrodin in vivo reduces cerebral edema and preserves nerve function after a TBI [70], while the knockdown of Sirt3 in the microglia eliminates the inhibitory effects of gastrodin on cell apoptosis and microglia activation following a TBI [53]. Furthermore, it has been reported that the upregulation of microglial Sirt3 expression promotes mitochondrial antioxidant enzyme function and reduces microglial senescence [71].

In conclusion, microglia activation and the inflammation that it causes can harm the CNS. One possible treatment approach is to use Sirt3 to inhibit hyperactive microglia.

## 4. Sirt3 and Neurodegenerative Diseases

With respect to the CNS, neurodegenerative diseases are referred to as the general term for illnesses resulting from the progressive chronic degeneration of the CNS tissue. The degenerative diseases of the CNS, such as AD, PD, HD, ALS, and MS, are regarded as the more harmful neurological diseases. They have become a worldwide healthcare issue [72]. The degenerative diseases of the CNS are currently not completely curable and can only marginally be improved with medications. Therefore, in-depth research is needed on the pathogenesis of degenerative diseases for the purpose of improving their clinical diagnosis, as well as the treatment effect. Sirt3 plays a key part in the CNS and participates in the regulation of the physiological and pathological functions of various nerve cells. Many of these mechanisms are closely related to neurodegeneration, implying that Sirt3 is a key regulatory molecule in neurodegenerative diseases (Table 1).

### 4.1. Sirt3 and Alzheimer’s Disease

AD, also known as senile dementia, is a neuronal degenerative disease that occurs mainly in the elderly and is characterized clinically by cognitive dysfunction, progressive memory impairment, language impairment, and personality changes [103]. Studies have shown that the pathogenesis of AD involves mitochondrial dysfunction, which is caused by multiple determinants that ultimately lead to necrosis, neuronal degeneration, or apoptosis [1]. Sirt3, an important regulator of protein deacetylation in the cellular mitochondria, plays a key role in maintaining the functional integrity of the mitochondria and has therefore received increasing attention in the study of AD. The current mechanisms of Sirt3 in AD mainly include (1) increasing the ATP levels in the mitochondria and promoting mitochondrial biosynthesis [104], (2) activating and enhancing mitochondrial dynamics [105], and (3) counteracting oxidative stress and regulating neuronal excitability [106]. Consequently, Sirt3 plays a protective role in AD.

Notably, the neurotoxic effects of amyloid beta (Aβ) play a crucial role in the development of AD [107]. In cortical samples from AD patients, the expression of Sirt3 mRNA is lower than that in healthy people [76]. According to a past study, neuronal apoptosis occurred when Aβ was added to a primary neuronal culture model, which was reversed using the neuroprotective factor pituitary adenosine live cell peptide (PACAP), whose protective effect was associated with the activation of mitochondrial Sirt3 synthesis [108]. After the knockdown of Sirt3, PACAP-mediated neuroprotection was lost [109]. In addition, curcumin, which has neuroprotective effects, attenuated Aβ-induced neuronal metabolic dysfunction and improved cognitive performance in a mouse model of AD by increasing Sirt3 activity [79]. This suggests that Sirt3 may have a neuroprotective effect on AD by modulating Aβ.

In addition to Aβ deposition, apolipoprotein E4 (APOE4) is an important genetic factor associated with the late onset of AD. Impaired learning and memory were observed in APOE4 transgenic mice [110,111]. In a study on human beings, Sirt3 expression was downregulated in the cerebral cortex of the APOE4 group compared to those without APOE4 expression [76]. APOE4 expression triggers mitochondrial oxidative stress, reduces ATP synthesis, leads to mitochondrial dysfunction, and subsequently disrupts synaptic transmission and leads to the emergence of cognitive impairment [77]. Meanwhile, the overexpression of Sirt3 improves memory and learning in APOE4 transgenic mice [78], which may be related to the fact that Sirt3 improves the antioxidant capacity of the nerve cells [112]. By regulating APOE4 and by improving brain energy metabolism, Sirt3 again plays a role with neuroprotective effects.

Thus, Sirt3 is crucial in AD-related pathogenesis (Table 1). Overall, as the world’s aging population continues to evolve, the Sirt3-mediated protective mechanisms provide an adequate basis for Sirt3 as a therapeutic target for AD, which can effectively prevent AD, can reduce the social burden, can improve the quality of life, and will become a potential target for the treatment of neurodegenerative diseases.

### 4.2. Sirt3 and Parkinson’s Disease

As the second-most prevalent neurodegenerative condition in the world, PD typically affects people over the age of 65, significantly impairs their ability to move, and impacts the quality of their lives [113]. Numerous investigations have suggested that dopaminergic neuron denaturation and death in the substantia nigra can be caused by mitochondrial malfunction [114], oxidative stress [115], anomalies in the ubiquitin–protease system, and α-synuclein accumulation [116]. A large number of studies have started to place a great emphasis on the connection between Sirt3 and PD’s pathogenesis because of the significant roles that energy metabolism disorders, mitochondrial oxidative stress, and the PD susceptibility genes PARKIN and PINK1 play in maintaining mitochondrial homeostasis [89].

The pathogenesis of PD is significantly influenced by mitochondrial dysfunction. In rat models of PD, abnormally folded and aggregated α-synuclein activates oxidative stress, damaging the mitochondria, which, in turn, harms neurons [116]. Recently, it was reported that Sirt3 protects the neurons by stabilizing mitochondrial energy metabolism in PD [84]. Sirt3 attenuates the death of the nigra dopaminergic neurons by reducing the buildup of oxidative stress products by deacetylating SOD2 and ATP synthase β-subunit [82]. Another study found that theacrine, a purine alkaloid, inhibits ROS production by activating mitochondrial Sirt3 and that it ultimately inhibits the apoptosis of the dopaminergic neurons [88]. As claimed by some researchers, a Sirt3 knockdown significantly exacerbates the death of the neurons and increases α-synuclein accumulation, whereas Sirt3 overexpression substantially reduces apoptosis, enhances cell viability, blocks the accumulation of α-synuclein, and decreases ROS production [83,86].

**Figure 4:**
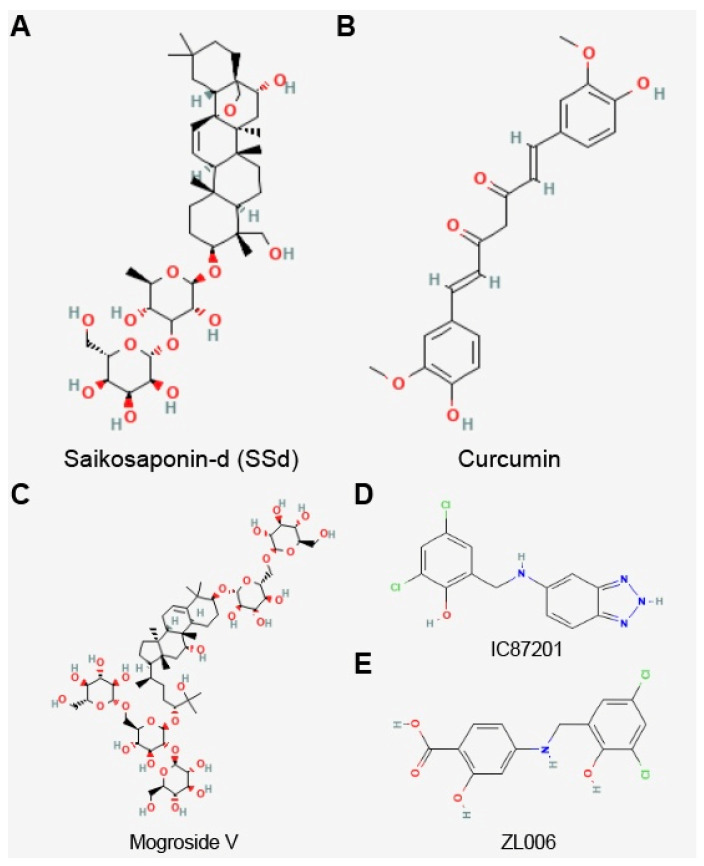
Chemical structure of Sirt3 activators. (**A**) Saikosaponin-d (SSd), (**B**) curcumin, (**C**) mogroside V, (**D**) IC87201, and (**E**) ZL006.

Currently, a connection between Sirt3 and PD has been discovered in a few drug studies. The neuroprotective effect of saikosaponin-d (SSd) [87], curcumin [85], and mogroside V [90] in PD cell models may be related to the reduction in the ROS level and the upregulation of Sirt3 expression (Figure 4). The compounds IC87201 and ZL006 activate the expression of Sirt3 through the inhibition of the interaction between postsynaptic density protein 95 (PSD-95) and neuronal nitric oxide synthases (nNOS), thereby mitigating the neuronal toxicity of PD [81]. In addition, melatonin mitigates PD dopaminergic neuronal damage by upregulating Sirt3 expression. Its mechanism of action is linked to its inhibition of microglia activation, alleviating inflammatory damage and oxidative stress.

The above findings reveal that Sirt3 has a certain relationship with the occurrence of PD, and Sirt3 can become a new target for PD therapeutic interventions; however, the investigation of the molecular mechanism of Sirt3’s specific role in PD development is not profound enough. Therefore, further studying the protective mechanism of Sirt3 in PD is crucial.

### 4.3. Sirt3 and Huntington’s Disease

HD is a rare autosomal dominant hereditary neurodegenerative illness characterized by progressive aggravated extrapyramidal symptoms, cognitive impairment, behavioral problems, and persistent chorea-like movements. The loss of many spiny striatal efferent neurons in the basal ganglia region, which results in aberrant dopamine, glutamic acid, and γ-aminobutyric acid transmission, is the primary pathogenic manifestation of HD [117]. As abnormal Huntingtin (Htt), a protein associated with HD, formation occurs with polyglutamine, patients develop metabolic disorders which may be caused by mitochondrial dysfunction [118]. Sirt3 has been reported to control mitochondrial function and is linked to oxidative damage, and it may provide a new biological target for HD treatment [119].

Studies on animals and people have discovered that the use of mitochondrial-oriented antioxidants in the treatment of oxidative damage can affect the level of Sirt3 [96], indicating that changes in the level and/or activity of Sirt3 are responses to significant oxidative damage [8,120]. A clear example is that the expression level of Sirt3 in the neurons with abnormal Htt in HD models is significantly reduced [91]. Additionally, trans-ε-viniferin can maintain the expression of Sirt3 in the cells, mediate the activation of AMPK and SOD2, alleviate the accumulation of ROS in the cells, promote the biogenesis of the mitochondria, improve the survival rate of the HD striatal cells, and produce neuroprotective effects [91,121]. Moreover, when using the HD mouse model induced with 3-nitropropionic acid (3-NP), it was found that Sirt3 knockout mice are more susceptible to the toxic effect of 3-NP than wild-type mice, further pointing out that Sirt3 might be an essential target in HD therapy [95].

This suggests that Sirt3 has a certain relationship with the occurrence of HD; however, clarifying the function of Sirt3 still necessitates more in-depth research and exploration. The study of Sirt3 will offer a novel approach for HD treatment due to the imbalance of Sirt3 expression.

### 4.4. Sirt3 and Amyotrophic Lateral Sclerosis

The majority of ALS patients die of respiratory failure or paralysis three to five years after the onset of symptoms [122]. ALS is a progressive lesion that causes motor neuron destruction in the anterior horn of the spinal cord [123]. The currently recognized pathogenesis of ALS consists of prion-like proliferation, an imbalance of protein homeostasis in the CNS, mitochondrial dysfunction, the spread of abnormal proteins, glutamate-mediated excitatory neurotoxicity, intraneuronal substance transport disorders, RNA metabolic disorders, and the abnormal apoptosis of the neurons [124]. The metabolic master regulator PGC-1α, a moderator of ALS in humans and model species, regulates Sirt3 expression [125]. Sirt3 reverses the abnormal metabolic patterns in the ALS motor neurons by acting as a mitochondrial deacetylase in ALS patients, preserving mitochondrial function and integrity [126].

Recent research has shown that boosting NAD^+^ levels, Sirt3 activity, and antioxidant defenses may be effective treatments for ALS [127,128]. The first evidence of a protective function of the Sirt3 single-nucleotide polymorphism rs4980329 in ALS came from a genetic investigation [129]. Mutations in the SOD1 gene can result in the hereditary neurodegenerative disease ALS [130] since it causes the shortening of the mitochondria, as well as an increase in rounded, fragmented mitochondria, affecting transport and ultimately leading to motor neuron death in the spinal cord [131]. Sirt3 can restore neuronal mitochondrial fragmentation and transport disorders caused by SOD1 mutations to a certain extent, reducing neuronal death and protecting against mitochondrial alterations in the SOD1-mutant neurons [97]. Moreover, Sirt3 can effectively antagonize SOD1-mutant astrocyte-mediated motor neuron damage, providing a new direction for the treatment of ALS [101].

At present, there is no effective cure for ALS, and the treatment is currently based on delaying the disease’s progress and improving the patient’s quality of life. With the deepening of the study of the Sirt3 mechanism, plenty of investigations have proven that Sirt3 is closely associated with ALS. Even though the specific relationship cannot be determined, it also provides a new direction for Sirt3-targeted formulations for ALS patients’ treatment.

### 4.5. Sirt3 and Multiple Sclerosis

MS is an autoimmune-mediated chronic inflammatory disease based on demyelinating CNS lesions with the pathological features of alternating relapses and remissions and the progressive loss of the neuronal myelin sheaths [132,133]. MS mainly develops in young adults, and elderly patients tend to have a progressive disease [134]. Previous research has shown that postmortem MS brain lesions and the experimental autoimmune encephalomyelitis (EAE) model both exhibit reduced Sirt2 expression [135]. Similar results were found for Sirt3, which exhibits lower expression in the postmortem brain tissues of MS patients [136].

A recent study showed that honokiol, a Sirt3 activator, protects C57BL/6 mice against EAE and that this protection is linked to a decrease in demyelination [137]. In addition, it is thought that the Sirt3 enzyme contributes to the development of myelin sheaths around mouse brain neurons. According to these preliminary findings, Sirt3 enzymes may become prospective therapeutic targets for the treatment of mitochondrial diseases, such as MS [138].

MS is associated with systemic immune disorders, the etiology of which is unclear. There are currently few studies on MS and Sirt3 (Table 1). Future studies on MS can further study the function of Sirt3 in MS [139], which will help to better understand the relationship between Sirt3 and MS and to discover new biomarkers, providing a new direction for clinical treatment.

## 5. Conclusions and Perspective

Since its discovery, the sirtuin family has been of wide concern and has been widely valued by the academic community. In recent years, the understanding of the sirtuin family has not remained on the simple description of “NAD^+^ dependent deacetylase”, and researchers have paid a considerable amount of attention to the molecular structure of each enzyme in this particular family, their distribution in the cells and tissues, their roles in biological processes, and their molecular mechanisms. In the past, Sirt1 was the focus of the majority of the studies on the sirtuin family. Numerous studies have lately shown that Sirt3 has special benefits and potential in neurodegenerative disorders. More and more research results have proven that Sirt3 has a unique role and potential in neurological diseases owing to its regulation of oxidative stress and energy metabolism, offering new treatment ideas for neurodegenerative diseases. Despite the fact that the relationship between Sirt3 and neurodegenerative diseases has achieved certain results, there are still a large number of controversial issues that urgently need to be resolved.

First, additional investigation is required to clarify the molecular mechanism by which Sirt3 functions as a protective factor in various neurodegenerative disorders. This will help to provide more sufficient evidence that Sirt3 acts as a target for the treatment of related neurological disorders. Second, on this basis, finding safe and reliable Sirt3 agonists and substrates for Sirt3 action will help in providing a deeper understanding of Sirt3′s protective role in neurodegenerative disorders, which is also an important direction for future research. Third, in order to apply Sirt3 to the clinic, for it to become a target for clinical interventions, and to exert its maximum value, emphasizing the significance of animal models in the study of the molecular mechanism and biological role of Sirt3 is necessary. Finally, animal models also need to further study the possible negative effects of Sirt3.

Currently, there is no Sirt3 activator drug candidate used in clinical practice for the treatment of neurodegenerative diseases. However, the clinical phase IV trial of silybin (a Sirt3 agonist) on hypertensive patients was completed in 2018. Based on the current research progress, we have reason to believe that the continuous in-depth study of the neuroprotective mechanism of Sirt3 will provide new means for neurodegenerative disorder treatment and prevention, and it is likewise expected to bring new hope to the treatment of related neurological diseases.

## Figures and Tables

**Table 1:** Sirt3′s roles in neurodegenerative diseases.

Neurodegenerative Disease	Mechanism	Experimental Setting	Research
AD	ROS in mitochondria increase Sirt3 expression.	Cell model	[73]
	Pharmacological enhancement of mitochondrial ROS increases the expression of Sirt3 in primary hippocampal cultures.	AD mouse model and cell model	[74]
	PACAP stimulates the production of mitochondrial Sirt3 and reduces neuronal death.	Postmortem human tissue, triple transgenic mouse model, and cell model	[75]
	Amyloid-β increases levels of total tau and acetylated tau through its modulation of Sirt3.	Postmortem human tissue	[76]
	APOE4 reduces ATP production by modulating the PGC-1α-Sirt3 signaling pathway, triggering mitochondrial oxidative stress and disrupting synaptic function.	Postmortem human tissue	[77]
	Sirt3 may mediate the neuroprotection of ketones by increasing neuronal energy metabolism.	APOE4 mouse model	[78]
	Alleviation of Aβ 42-induced neuronal metabolic dysfunction occurs via the THRB/Sirt3 axis and improves cognition.	APP_TG_ mouse model	[79]
	Activation of mitophagy and mitochondrial unfolded protein response occurs.	APP/PS1 mouse model	[80]
PD	IC87201 and ZL006 reduce ROS production and improve mitochondrial dysfunction by increasing the expression of Sirt3 after MPP+ exposure.	MPP+-induced primary cortical neuron cell models	[81]
	Sirt3 has a possible role in MPTP-induced neurodegeneration by preserving the free radical scavenging capacity of mitochondria.	Sirt3 null mouse model	[82]
	Sirt3 overexpression dramatically increases cell viability, decreases cell apoptosis, prevents the accumulation of α-synuclein, suppresses the reduction of SOD and glutathione, decreases ROS generation, and alleviates MMP collapse induced by rotenone.	PD cell model	[83]
	Sirt3 rescues neurons through the stabilization of mitochondrial biogenetics.	Virally expressed mutant α-synuclein rat model of parkinsonism	[84]
	Curcumin lowers ROS levels in SH-SY5Y cells and upregulates Sirt3 expression.	SH-SY5Y cell models	[85]
	miR-494-3p downregulation increases Sirt3 expression, reduces oxidative stress, and improves dyskinesia.	MPTP-induced PD mouse model and SH-SY5Y cell model	[86]
	Saikosaponin-d exerts a neuroprotective effect by upregulating Sirt3 expression and alleviating oxidative stress damage.	MPP+-induced SH-SY5Y cell models	[87]
	Sirt3 mediates SOD2 deacetylation to reduce ROS accumulation and to restore mitochondrial function, thereby preventing apoptosis.	6-OHDA-treated rat, MPTP-treated mouse, and zebrafish models	[88]
	Regulation of Sirt3 in mitochondrial functions and oxidative stress occurs in PD.	Sirt3 null mouse and PD mouse models	[89]
	Upregulated Sirt3 mitigates the protective effect of mitochondrial dysfunction on neuronal damage.	SH-SY5Y cell models	[90]
HD	Knockdown of Sirt3 significantly inhibits viniferin-mediated AMP-activated kinase activation and diminishes the neuroprotective effects of viniferin.	Mutant HTT cell model	[91]
	Increased Sirt3 levels and/or activity reduce oxidative damage.	Cell model, HD knockin mouse model, and Huntington’s disease transgenic (YAC128) mouse model	[92,93,94]
	Sirt3 protects neurons against metabolic and oxidative stress by reducing mitochondrial superoxide levels, stabilizing cellular and mitochondrial Ca2+ homeostasis, and inhibiting mitochondrial membrane permeability transition pore formation to prevent apoptosis.	Cell model and HD mouse model	[95]
	Sirt3 overexpression promotes the antioxidant effect of cells expressing mutant HTT, leading to enhanced mitochondrial function and balanced dynamics.	Postmortem human tissue and primary striatal neuron cell model	[96]
ALS	Sirt3 protects against mitochondrial fragmentation and neuronal cell death with mutant SOD1 (G93A).	SOD1^G93A^ transgenic mouse model and primary cortical neuronal cell model	[97]
	Overexpression of Sirt3 increases NADPH levels and protects from oxidative-stress-induced cell death.	Sirt3 mouse model	[98]
	Grape wine polyphenols prevent axonal apoptosis and act via mitochondrial Sirt3 activation in axons.	Primary cortical neuronal cell model	[99]
	Sirt3 can restore neuronal mitochondrial fragmentation and transport disorders, reducing neuronal death, and protects against mitochondrial alterations.	SOD1-mutant cell model	[100,101]
MS	The EA protects muscle tissue from cuprizone-induced demyelination by overexpressing Sirt3 to protect mitochondria and to reduce oxidative stress.	Mouse model	[102]

## Data Availability

No new data were created or analyzed in this study. Data sharing is not applicable to this article.
